# Assessment of PTeye™ versus FLUOBEAM® LX for parathyroid adenomas: a pilot case–control study

**DOI:** 10.1007/s13304-025-02334-7

**Published:** 2025-07-29

**Authors:** Theodosios Papavramidis, Angeliki Chorti, Sohail Bakkar

**Affiliations:** 1 1 st Propedeutic Department of Surgery, AHEPA University Hospital, Aristotle University of Thessaloniki, St Kiriakidi 1, 54636 Thessaloniki, Greece; 2 Unit of Minimally Invasive Endocrine Surgery, Kyanous Stavros, Thessaloniki, Greece; 3 Department of Surgery, Faculty of Medicine, the Hashemite University, Zarqa, 13131 Jordan

**Keywords:** Parathyroid adenoma, PTeye, FLUOBEAM, Adenoma identification

## Abstract

Identifying the parathyroids is compulsory for success of parathyroidectomy for parathyroid adenoma. The aim of the present study is to evaluate and compare the efficacy of PTeye™ and FLUOBEAM® LX in identifying parathyroid adenomas. Patients undergoing parathyroidectomy due to a parathyroid adenoma were enrolled prospectively in this study and were randomly included to Group A (PTeye™) or Group B (FLUOBEAM® LX). After intraoperative identification of parathyroid adenomas and before tissue dissection (minute 0), we evaluated the efficacy of both devices in confirming the adenomas. We re-evaluated devices’ efficacy in minutes 1, 3 and 5 during tissue dissection and before adenoma excision. All PAs were confirmed and identified with PTeye™, while FLUOBEAM® LX could not identify 3/20 adenomas (15%). PTeye™ confirmed parathyroid tissue in less than 1 min in 13 cases (65%), in < 3 min in 7 (35%), whereas FLUOBEAM® LX identified 4 adenomas in < 3 min (20%), in < 5 min 9 adenomas (60%) and > 5 min in 4 (20%). PTeye™ and FLUOBEAM® LX are both useful tools in confirming parathyroid tissue intraoperatively. PTeye™ confirmed the suspected adenoma earlier before tissue dissection, while FLUOBEAM® LX demands tissue dissection as it identifies the normal parathyroid tissue.

## Introduction

Parathyroid adenoma (PA) is the most common cause of primary hyperparathyroidism (HPT), identified and diagnosed in an increasing proportion of people worldwide[1]. Parathyroidectomy is the conventional treatment approach for parathyroid adenomas (PAs)[1]. Identifying the parathyroid glands is essential for the success of parathyroidectomy. This can be a challenging and time-consuming endeavor for all surgeons [2–4].

Intraoperative parathyroid hormone assays are the cornerstone for the confirmation of PA excision, but it really increases the operation time. Thus, there is a compulsion for a real-time tool for identifying parathyroid glands[5]. Near- infrared autofluorescence (NIRAF) has been proposed as a tool for label-free identification of parathyroid glands intraoperatively[6]. Two approaches are available to surgeons to perform near-infrared autofluorescence: (i) Camera-based systems (CBS), such as FLUOBEAM® LX (Fluoptics, Grenoble, France), and (ii) Probe-based systems such as PTeye™ (Medtronic, Minneapolis, MN) [5].

The present study aims to evaluate the performance of two novel devices (PTeye™ and FLUOBEAM® LX) in identifying PAs.

## Methods

### Data collection

This is a single-center prospective case–control study. From September to December 2024, patients undergoing parathyroidectomy for single PA, to Euromedica Kyanous Stavros Clinic, were included and equally assigned in either PTeye group or Fluobeam group. Exclusion criteria were: age under 18 years old, secondary and tertiary HPT, parathyroid hyperplasia, parathyroid carcinomas. Preoperative evaluation included laboratory examination and exact localization of the adenoma by two independent imaging modalities (ultrasound, Tc-99 sestamibi scintigraphy, magnetic resonance imaging, 4D- computed tomography).

Minimally invasive single gland parathyroidectomy was performed by the same high-volume endocrine surgeon (> 100 thyroidectomies and parathyroidectomies per year). A small neck incision (2 cm) was perfomed and blunt dissection of strap muscles followed. The thyroid lobe was carefully retracted according to preoperative localization of PA. Before dissection of the adenoma, PTeye™ or FLUOBEAM® LX were applied for the intraoperative identification of PA. If the device failed to identify the adenoma, dissection of the surrounding tissue was performed and the NIRAF device was re-applied. All parathyroidectomies were guided by intraoperative quick PTH assays (parathyroid hormone assays).

Clinicopathological data (age, gender, biochemical data, parathyroid pathology and PA identification time have been recorded (Table 1). PA identification time was defined as the time elapsed between exposure of the thyroid and the NIRAF identification of PA. Identification times were classified in 4 categories: (a)under 1 min, (b) under 3 min, (c) under 5 min and (d) above 5 min.

**Table 1:** Epidemiologic and biochemical characteristics of both study groups

	Group PTeye™ n = 20	Group FLUOBEAM® LX n = 20	p-value
Age (years)	54.2 ± 11.4	53.2 ± 12.4	0.79
Sex (Female/Male)	16/4	15/5	
PTH (pg/ml (SD)) preoperative postoperative	108 (IQR = 52.2) 15.50 (IQR = 7.9)	110 (IQR = 41) 19.30 (IQR = 9.1)	0.8 0.23
Calcium (mg/ml) (SD) preoperative postoperative	10.97 (IQR = 1.13) 9.12 (IQR = 1.16)	10.90 (IQR = 2.9) 9.4 (IQR = 1.13)	0.77 0.16
Parathyroid weight (g)	0.90 (IQR = 0.83)	0.63 (IQR = 0.5)	0.19
Parathyroid maximal dimension (cm)	1.75(IQR = 1.18)	1.60 (IQR = 0.75)	0.2

Data collection and analysis were performed according to the institute’s scientific policy that ensures maintaining data confidentiality and using it for scientific purposes only, and the ethical standards of the Helsinki declaration 1975 and its later amendments and comparable ethical standards. Institutional review board (IRB) approval (No. 5/3.9.24) was obtained. Patient informed consent was obtained. This study has been registered to Clinicaltrials.gov (registration number: NCT06788223).

### Statistics

Quantitative variables following a normal distribution were reported as means ± standard deviation, while those not following a normal distribution were reported as medians with interquartile ranges. Qualitative variables were reported as frequencies. Sample normality was tested using the Shapiro–Wilk test. If the continuous variables didn’t follow a normal distribution, non-parametrical tests were used. To compare the median values of the quantitative variables in the categories of binary categorical variables, the Mann–Whitney test was used and for mean values in normal distribution, independent sample t-test was applied. Finally, with the X^2^ test (between categorical variables), possible correlations and dependencies between the variables were investigated. The statistical processing of the sample was done using the statistical package “Statistical Package for the Social Sciences (SPSS)” version 27 (IBM), (SPSS Inc., Chicago, IL, USA). In all cases, a *p*-value < 0.05 was set as the level of statistical significance for a two-tailed test.

## Results

In this study, 40 patients were enrolled and equally assigned to group PTeye and group FLUOBEAM. No differences in epidemiological and biochemical characteristics of both groups were observed (Table 1).

PTeye™ succeeded in identifying and confirming all PAs (20/20, 100%), while FLUOBEAM® LX could not identify 3/20 adenomas (15%).

**Fig. 1:**
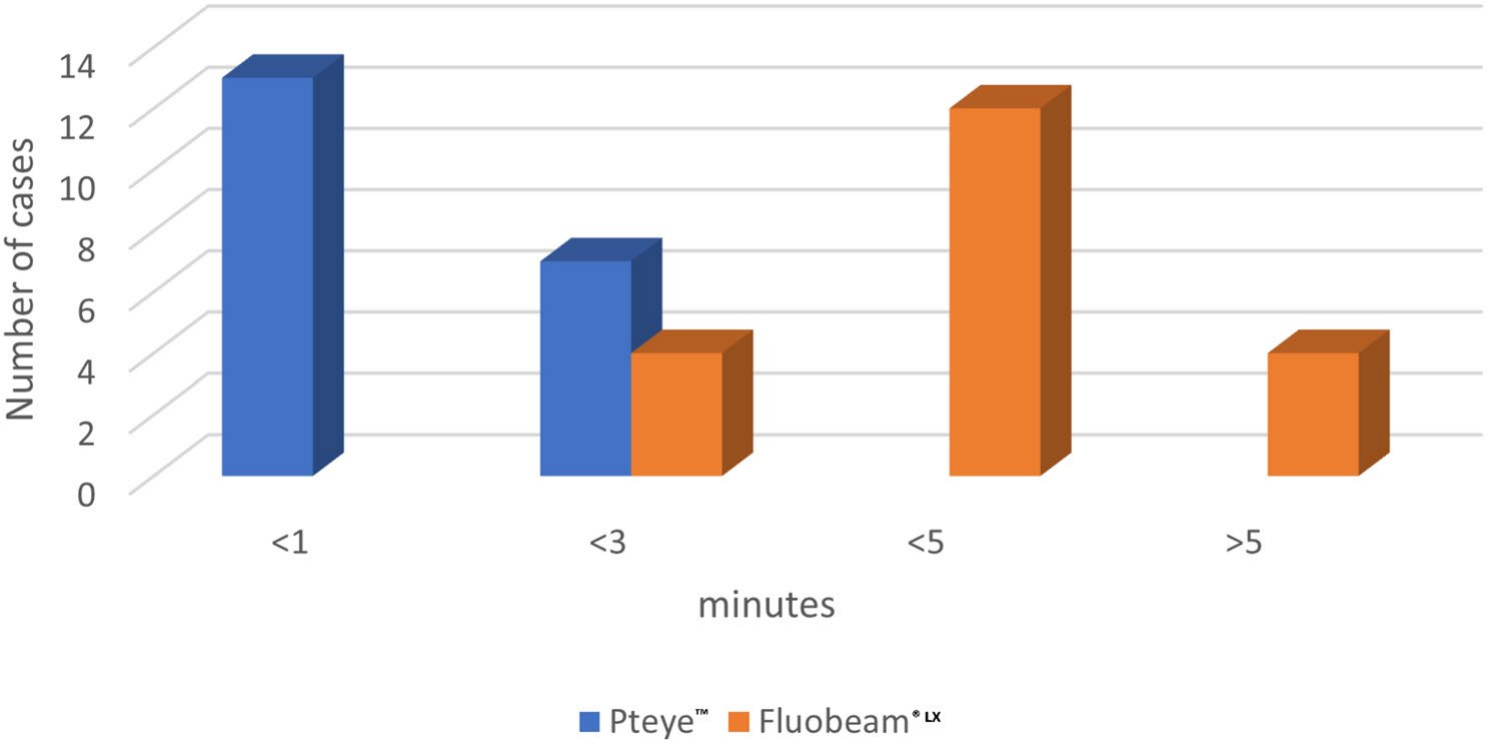
Adenomas detected in each time frame for each method

Regarding identification times, PTeye™ confirmed parathyroid tissue existence in less than 1 min in 13 cases (65%) and in < 3 min in 7 (35%), whereas FLUOBEAM® LX identified 4 adenomas in < 3 min (20%), in < 5 min 9 adenomas (60%) and > 5 min 4 adenomas (20%). Figure 1 summarizes the above data.

Neither the identification rate nor identification times showed statistically significant correlation with the clinicopathological data.

## Discussion

Our study confirms the utilization of near-infrared autofluorescence technology as an intraoperative tool for parathyroid adenoma identification. Τhere are nuances in the applications of NIRAF technologies in parathyroidectomy. Those technologies may be a great adjunct to non-specialized surgeon in achieving optimal results. Moreover, it can be useful in cases of recurrent operations that the anatomy is altered.

The utility of probe-based systems (PBS) such as PTeye™ in recognizing parathyroid adenomas has been already reported[7, 8]. Thomas et al. demonstrated that PTeye™ device had a sensitivity of 97% and a specificity of 84.2%, with positive predictive value of 91.4% and negative predictive value of 94.1%[8]. In our study, the sensitivity for PTeye™ was 100% and for FLUOBEAM® LX 85%, but due to lack of true negative data, the other parameters could not be calculated.

We demonstrated that probe-based systems (PBS) show an advantage over camera-based systems (CBS) concerning the speed of definitive recognition of pathologic parathyroids. In classical operation, once the suspicious structure is palpated, there is meticulous dissection in order to visually identify PA. Nowadays, we try to further identify PAs by NIRAF [2, 5]. Therefore, future surgeons can consider NIRAF in lieu of frozen section biopsy.

Potential challenges hindering the effectiveness of NIRAF imaging in parathyroidectomy are the heterogenous autofluorescence pattern of PAs and the limited depth-penetration of near-infrared light waves [9]. The major issue for NIRAF is the abnormally lower auto-fluorescence demonstrated by PAs compared to normal parathyroids [7, 8, 10]. Parathyroid adenomas show a heterogeneous autofluorescence pattern. A specific region on the PA, called “cap” seemed to be 28% more fluorescent than the rest of the adenoma[11]. This specific rim of PA, “cap”, is present in the majority of parathyroid adenomas (various proportions in literature, ranging from 64.3% to 73.9%) [11, 12]. While, CBS seem to be greatly influenced by the aforementioned phenomenon (meticulous dissection of the pathologic gland is necessary, in order to identify the “cap” and achieve a positive auto fluorescent signal), PBS seems not to be influenced[8]. The present study enforces the above-mentioned since all PAs were identified with the probe in less than 3 min, while with the camera 80% PAs were identified in more than 3 min.

There are two additional limitations related to NIRAF CBS: (i) the wide view of the operative field, makes their use difficult with small incisions (increased background noise from the skin), and (ii) the penetrating ability of the near-infrared laser, limits the depth of parathyroid identification. Only 26% of PAs were identified by NIRAF without dissection[10]. Conversely, the probe can be applied on any palpated structure (one or more times) without any dissection (increasing this way the accessibility and the speed of the method). PBS system can be applied with accurate results even with no direct contact with the tissue[7].

Despite the small sample size, we can conclude that PTeye™ and FLUOBEAM® LX are intraoperative tools in confirming parathyroid tissue. PTeye™ confirmed the suspected adenoma earlier and without dissection, while FLUOBEAM® LX demands tissue dissection as it identifies the normal parathyroid tissue of the “cap”.

## Data Availability

The data will be provided under request.
